# Potential of Immunoglobulin A to Prevent Allergic Asthma

**DOI:** 10.1155/2013/542091

**Published:** 2013-04-11

**Authors:** Anouk K. Gloudemans, Bart N. Lambrecht, Hermelijn H. Smits

**Affiliations:** ^1^Department of Pulmonology, Erasmus Medical Center, Rotterdam, The Netherlands; ^2^Leiden Immunoparasitology Group, Department of Parasitology, Leiden University Medical Center, Albinusdreef 2, P4-37A, 2333 ZA Leiden, The Netherlands; ^3^Laboratory of Immunoregulation and Mucosal Immunology, Department of Molecular Biomedical Research, VIB, Technologiepark 927, 9052 Ghent, Belgium

## Abstract

Allergic asthma is characterized by bronchial hyperresponsiveness, a defective barrier function, and eosinophilic lower airway inflammation in response to allergens. The inflammation is dominated by Th2 cells and IgE molecules and supplemented with Th17 cells in severe asthma. In contrast, in healthy individuals, allergen-specific IgA and IgG4 molecules are found but no IgE, and their T cells fail to proliferate in response to allergens, probably because of the development of regulatory processes that actively suppress responses to allergens. The presence of allergen-specific secretory IgA has drawn little attention so far, although a few epidemiological studies point at a reverse association between IgA levels and the incidence of allergic airway disease. This review highlights the latest literature on the role of mucosal IgA in protection against allergic airway disease, the mechanisms described to induce secretory IgA, and the role of (mucosal) dendritic cells in this process. Finally, we discuss how this information can be used to translate into the development of new therapies for allergic diseases based on, or supplemented with, IgA boosting strategies.

## 1. Introduction 

Allergic asthma is a major health problem worldwide, causing episodes of wheezing, coughing, and breathlessness in susceptible individuals after repeated inhalation of harmless environmental allergens, such as house-dust mites (HDMs), molds, plant pollen, and animal dander [1, 2]. Currently approximately 300 million people worldwide suffer from asthma, with estimates suggesting that asthma prevalence increases globally by 50% every decade. Prevalence has reached a peak in developed countries, but rates are rising in developing regions (Africa, Latin America, and parts of Asia) as they become more westernized. This has reduced the global differences in prevalence; however, the global burden of asthma and allergies continues to rise, and new therapies are warranted [3, 4].

Interestingly, negative associations are found between the prevalence of allergic asthma and growing up on traditional European farms or rural tropical areas, usually exposed to higher ambient concentrations of microbial pollutants or higher rates of parasitic infections [5, 6]. Therefore, it was suggested that a reduced microbial exposure during childhood, due to changes in lifestyle, vaccination patterns, and/or improved hygiene, has contributed to the global increases in hyperinflammatory diseases. Insufficient microbial exposure may result in deficient maturation of the regulatory arm of the immune system, causing a disbalance of the immune system, allowing for uncontrolled expression of inflammatory responses against innocuous antigens later in life (“hygiene hypothesis”) [7]. 

Currently used medication against asthma is aimed at symptom relieve and does not restore this immune disbalance. As a consequence, treatment is chronic and sometimes resulting in severe side effects. New therapies should focus on reducing inflammatory responses against allergens at an early age preventing the onset of structural damage and changes to the lungs, by targeting natural tolerizing mechanisms as found in healthy individuals. In this review, we will focus on one of these mechanisms and describe the potential of inducing IgA responses by modulation of dendritic cell function and controlling unwanted allergic responses. 

## 2. Immune Responses against Allergens

### 2.1. Inflammatory Reactions against Inhaled Allergens in Allergic Asthma: Th2 Cells and IgE

Allergic asthma is a chronic inflammation of the airways controlled by effector Th2 cells and characterized by eosinophilic airway inflammation and high levels of allergen-specific IgE antibodies, hallmarks of a persistent Th2 response [2] (Figure 3(a)). Upon encounter with the allergen, effector responses can be divided into immediate and late phase reactions. The immediate allergic inflammatory reaction is initiated by crosslinking of IgE molecules which are bound to IgE receptors on basophils and mast cells. As a result, these cells will degranulate and release preformed mediators from vesicles or secrete cytokines (IL-6, TNF*α*, MIP1*α*) [9], causing immediate vascular permeability, blood vessel dilation, bronchoconstriction, and smooth muscle contraction [10]. This immediate reaction may be followed by the late phase response, initiated by inflammatory cytokines and type 2 cytokines, such as IL-4, IL-5, Il-9, and IL-13, which recruit and activate eosinophils and basophils and induce gobleT-Cell metaplasia and overproduction of mucus [11, 12]. In severe forms of asthma, also Th17 cells are found, which enhance the effects of the Th2 cytokines and recruit neutrophils and other inflammatory leukocytes [13–15]. In addition to distortion of immunological pathways during allergen sensitization and challenge, also aberrant structural airway remodeling is involved in the development of asthma. Some groups have even suggested that the airway structural changes occur before the deranged immune response is present. Indeed, basement membrane thickening is detectable in children younger than three years old with persistent wheezing before the diagnosis of asthma [16, 17]. Airway remodeling includes marked changes in the airway wall, like epithelial injury, extracellular matrix deposition under the epithelial basal membrane, gobleT-Cell hyperplasia, and increased smooth muscle mass. These changes lead to a defective physical and functional barrier of the airway epithelium in severe asthma. Various studies point at dysfunctional injury/repair mechanisms in response to damaging stimuli and/or respiratory viruses in asthmatics, which may only be in part explained by allergic airways inflammation [18, 19].

Dendritic cells (DCs) play a crucial role in the described processes leading to asthma pathogenesis (Figure 3(a)). Immature DCs reside in peripheral and mucosal tissues, such as the lungs, where they continuously sample the environment for foreign soluble antigens and small particles, including inhaled allergens [20, 21]. DCs express different types of pattern recognition receptors (PRRs), such as toll-like receptors (TLRs), NOD-like receptors, and C-type lectin receptors, that allow the recognition of different classes of molecules broadly shared by pathogens (pathogen-associated molecular patterns (PAMPs)) [22, 23]. Upon encounter of danger signals, DCs become activated and migrate to the draining lymph nodes [24], where they activate antigen-specific naïve Th cells and drive their development in effector T helper cells, such as Th1, Th2, Th17, or regulatory T (Treg) cells [25, 26]. Various studies have demonstrated that DCs are necessary for inducing allergic sensitization [27], for driving the development of Th2 immunity and eosinophilia [28–30], and are crucial for maintaining the inflammatory processes in the airways as well as bronchial hyperreactivity and chronic airway remodeling [31, 32]. After allergen uptake, the function of DCs is strongly influenced by signals encountered during their stay in the peripheral tissues, which can include microbial signals induced by the ligation of pattern recognition receptors (PRRs) on the DCs or alarming signals from structural cells like local epithelial cells of the airways [33–35]. Crosstalk between airway epithelial cells and DCs may form a critical link for the induction and continuation of allergic inflammation in the lungs as several EC-derived molecules can influence DC migration, differentiation, and function [20, 33, 36].

### 2.2. “Tolerizing” Immune Responses to Allergens in Healthy Individuals: Treg Cells and IgA

In healthy individuals, T-cell responses to allergens are commonly observed, yet are usually dominated by anergy or by regulatory T (Treg) cells that can suppress various effector Th cell subsets [37, 38]. Allergen-specific Treg cells can suppress Th2 cells by cell-cell contact or release of the anti-inflammatory and immunoregulatory cytokines IL-10 and transforming growth factor (TGF)-*β*. In almost all patients with asthma, one can find the counterregulatory Treg cells, but these fail to or insufficiently suppress allergic inflammation [39]. It has therefore been suggested that asthma may result from aberrant of defective Treg mechanisms.

Humoral responses of healthy individuals consist of mainly low IgG1, IgG4, and secretory IgA (sIgA) antibodies to allergens in the presence or absence of low amounts of IgE [37, 40, 41]. Although the presence of allergen-specific IgA has drawn relatively little attention so far, it is still unclear what its relative role is in the protection (or exacerbation) of allergic disease [42]. Although most individuals with immunoglobulin A (IgA) deficiency are asymptomatic, allergic disorders appear to be more common among patients with IgA deficiency [43]. Indeed, Balzar et al. found lower IgA levels in bronchoalveolar lavage of severe asthmatics than in healthy subjects, which correlated with lung function and asthma symptoms [44]. In contrast, high salivary secretory IgA levels were associated with less development of allergic symptoms in sensitized Swedish children [45]. Furthermore, high levels of specific IgA antibodies in salivary of sensitized infants were associated with significantly less late-onset wheezing [46]. In addition, allergic patients who naturally develop tolerance responses towards cow's milk concomitantly undergo a shift towards IgA dominance in serum [47]. Moreover, in an experimental setting, Schwarze was able to protect mice against the development of eosinophilic airway inflammation and hyperresponsiveness by treating with antigen-specific IgA during challenge [48]. 

Taken together, these data show an inverse relationship between IgA and allergy development, suggesting a protective role for IgA in allergic diseases such as asthma. 

## 3. Immunoglobulin A Antibodies and Its Functions

### 3.1. Isoforms and Receptors

**Figure 1:**
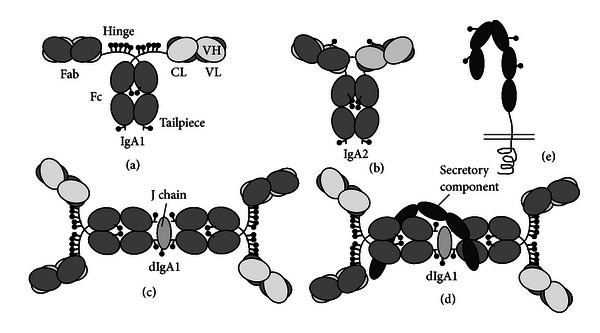
Human IgA structure. Schematic diagrams of (a) IgA subclass 1 (IgA1), (b) IgA2, (c) dimeric IgA1 (dIgA1), (d) secretory IgA1, and (e) polymeric immunoglobulin receptor (pIgR) (adapted from [8]).

The antibody IgA can occur as a monomer (Figures 1(a) and 1(b)), but also in dimeric or even polymeric forms through interactions with the joining chain (J-chain) (Figure 1(c)). All these different forms are mainly found in the circulation, while secretory IgA (sIgA) is only found at mucosal surfaces and is generated by the binding of dimeric IgA via the J-chain to the polymeric immunoglobulin receptor (pIgR) at the basolateral side of the epithelium which is subsequently transported to the luminal side (Figures 1(d) and 1(e)). Here, IgA is released at the mucosal surface (lumen) by cleavage from the pIgR. In this process part of the pIgR, called the secretory component (SC), remains attached to the IgA molecule, and together they form the molecule secretory IgA (SIgA). Mouse and human IgA biology differ in several aspects. In human serum, IgA occurs mainly in a monomeric form, while in mice polymeric IgA is the main isotype in serum. Furthermore, human IgA, but not mouse IgA, is divided into closely related subclasses, IgA1 and IgA2, of which the later one is less susceptible for proteolytic degradation (Figures 1(a) and 1(b)). In serum, the subclass IgA1 is dominant, while in secretions the main isoform found is IgA2, although both IgA1 and IgA2 can be detected as SIgA [49]. 

IgA has been described to interact with various host receptors, that is, pIgR, Fc*α*RI (CD89), transferrin receptor (CD71), asialoglycoprotein receptor (ASGPR), and Fc*α*/*μ*R. The consequences after ligation are not very clear for most of these receptors. However, the Fc*α*RI allows both inhibitory and activating signals and therefore is considered to be important for the role of IgA in preserving homeostasis and tolerance at mucosal sites [8, 50]. Moreover, Fc*α*RI is the only IgA Fc receptor expressed on (blood) myeloid cells, including DCs, monocytes/macrophages, neutrophils, and eosinophils. In mucosal areas in steady state conditions, only few cells are positive for Fc*α*RI. Intriguingly, this receptor has not been identified in mice. Although this receptor is associated with an immunoreceptor tyrosine-based activation motifs (ITAM), its signaling can be activating as well as inhibitory. This depends on the ligand and subsequent configuration (involving Syk or SHP-1 phosphatase) of the ITAM, resulting in an activating or an inhibitory ITAM motif. The inhibitory ITAM (ITAMi) pathway takes place in the absence of receptor coaggregation and of an immunoreceptor tyrosine-based inhibitory motif (ITIM), which is known for inhibiting immune responses. All forms of IgA can ligate to Fc*α*RI, but they differ in their binding capacities. Monomeric IgA only binds with low affinity to the Fc*α*RI and activates the ITAMi, which does not lead to cell activation or degranulation/oxidative burst (in the case of granulocytes) [51]. In contrast, IgA complexes show a stronger binding and subsequent activating signal, resulting in cell activation [52, 53]. 

### 3.2. Effector Functions of IgA

IgA is classically known for neutralizing toxins and bacteria (viruses) at mucosal surfaces [54, 55], by interfering with their motility, by competing for epithelial adhesion sites, and by improving the viscoelastic properties of the airway secretions [56]. The SC protects SIgA from proteolytic degradation and is involved in establishing local interactions with bronchial mucus, thereby contributing to the “trapping” and removal of the antigen (“immune exclusion”) [57]. Interestingly, it has been suggested that IgA can also directly reduce inflammatory responses by inhibiting effector functions of inflammatory cells. For example, anti-Fc*α*RI Fab treatment, by initiating ITAMi signaling, suppressed manifestations of allergic asthma in Fc*α*RI transgenic mice immunized with anti-IgE immune complexes [58]. Triggering ITAMi signaling also prevented marked inflammation and leukocyte infiltration in kidney inflammation models such as glomerulonephritis [59]. Furthermore, *in vitro* crosslinking of Fc*α*RI on human monocyte-derived DCs leads to internalization of IgA complexes and antigen presentation, resulting in DC maturation and IL-10 production [60, 61]. (Serum) IgA ligation on monocytes also induces IL-10 expression [62] and inhibits inflammatory cytokine (IL-6 and TNF*α*) release [63, 64]. Importantly, IgA has only limited capacity to activate the complement system, in contrast to IgG and IgM. Furthermore, it can competitively block the IgG-mediated activation of complement [8, 65]. Of note, a few specific diseases are associated with an increase in serum IgA levels, often paralleled by IgA tissue deposition [66]. In IgA nephropathy, the formation of aggregated IgA immune complexes in the kidney causes severe inflammatory responses [67, 68]. However, there are indications that in these patients, glycosylation (e.g., sialylation) of the circulating IgA antibodies is abnormal, which may explain the pathogenic potential [53]. 

Collectively, these data suggest that under homeostatic conditions, secretory IgA contributes to the maintenance of mucosal tolerance by dampening immune responses. Therefore, IgA can have a role in preventing the development of hyperinflammatory responses towards environmental allergens that otherwise could cause allergic inflammation as observed in allergic rhinitis or asthma. 

## 4. Regulation of Immunoglobulin A Responses 

### 4.1. T-Cell-Dependent and T-Cell-Independent IgA Class Switching

**Figure 2:**
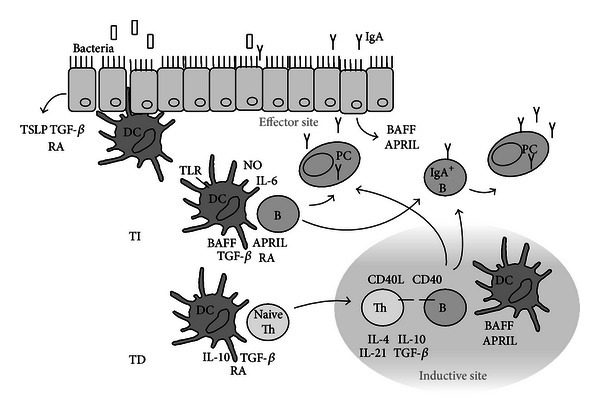
After conditioning by tissue-derived factors and TLR ligands, DCs activate T cells which provide for costimulatory signals (CD40-CD40L) and specific cytokines (TD), or DCs provide for alternative costimulatory signals, like BAFF and APRIL (TI) to initiate class switch recombination and expression of IgA by the mature B cells (IgA^+^ B cell). From the inductive site, the IgA^+^-B cells will migrate to the draining effector sites, where, in response to additional signals, they become plasma cells (PCs) and start to secrete IgA.

Humoral responses in the mouse are mediated by at least three different subpopulations of mature B cells. These B cells can acquire the expression of various antibody isotypes, including IgA, by undergoing class switch recombination (CSR). In contrast to other isotypes, IgA class switching can occur both via a conventional T-Cell-dependent (TD) pathway and an alternative T-cell-independent (TI) pathway (Figure 2). Follicular (or B-2) B cells, located in the spleen, lymph nodes, and the Peyer's Patches respond to T-cell-dependent antigens and can acquire the expression of various isotypes by undergoing class switch recombination (CSR). The resulting IgA^+^-B cells will migrate to the draining effector sites. TD class switch is induced by CD40-CD40L ligation and specific cytokines secreted by T cells as a result of activation by DCs or other APCs. The major cytokine signal for *α*-CSR is TGF-*β* with contributions from IL-2, IL-4, IL-5, IL-6, IL-10, and IL-21 [69–72] (Figure 2 TD pathway). 

Nonfollicular B cells, such as the splenic marginal zone B cells and the B-1 cells, which are mostly enriched in the peritoneal and pleural cavity and the lamina propria of the small and large intestines, primarily respond to T-cell-independent antigens and secrete natural or polyspecific antibodies [73]. The alternative TI pathway occurs locally at effector sites and is a much faster mechanism to generate IgA. TI class switching is induced independently of CD40-CD40L engagement and needs alternative costimulatory signals, such as B-cell activating factor of the TNF family (BAFF, also known as BLyS), a proliferation-inducing ligand (APRIL), retinoic acid (RA), TGF-*β*, nitric oxide (NO), and/or IL-6. These IgA costimulatory factors can be produced by both resident epithelial cells of mucosal organs and by local DCs. In fact, mucosal DCs, from Peyer's Patches (PPs), gut lamina propria [74], or lungs [75], are the primary APCs able to drive TI IgA class switching. Once CSR has taken place, most of these factors, including BAFF and APRIL, further enhance both TD and TI IgA responses by providing survival signals, and/or inducing plasma cell differentiation and IgA secretion, pointing at an additional role of structural cells and DCs at a later stage of IgA development [69–71] (Figure 2 TI pathway). 

It is increasingly clear that gut IgA-producing B cells can be both generated from follicular B cells or the B-1 cells by complementary pathways, requiring different signals to undergo IgA switching which links back to their capacity to respond to TD or TI antigens. For example, peritoneal B-1 cells more readily switch to IgA *in vitro* in response to BAFF, TLR ligation, and TGF-*β*, while follicular B cells or peritoneal B-2 cells require cytokines like IL-4, IL-5, or anti-IgD dextran [76]. Interestingly, B-1 cells can switch to all immunoglobulins *in vitro*, while *in vivo* studies with SCID mice or irradiated mice reconstituted with bone marrow or peritoneal cavity cells have suggested that B-1 cells preferentially switch to IgA [77, 78], where they accounted for most of the gut IgA plasma cells. However, studies in gnotobiotic allotype Ig chimeric mice (allowing the distinction between Abs derived from B1 and B2 cells based on different allotypes) suggested that in normal immunocompetent mice intestinal B-2 cells contributed for most of the IgA found in the gut in response to gut bacteria [79]. Importantly, also in the respiratory tract and their draining lymph nodes local B-1 cells have been demonstrated [80]; however, the respective role of B-1 or follicular B cells in the production of IgA has not been studied yet. The relative role of B-1 versus B-2 cells in IgA-mediated immunity is reviewed elsewhere [72].

Theoretically, it might be possible to induce *in situ* CSR of existing allergen-specific IgE^+^ B cells into IgA_2_^+^ cells. Because C_H_ *α*_2_ is the last exon located downstream from C_H_ *ε* in the human heavy chain locus, this may be the only alternative for CSR in IgE^+^ B cells. Shifting the allergen-specific antibody response from IgE to IgA2 would result in neutralization of allergen in the mucosal lumen, before it could interact with IgE, and could therefore constitute a therapeutic target. Although this has not been tested nor reported yet, based on the role of IL-21 and TGF-*β* in IgA class switching [81], they may contribute to induce IgA2 production in already class-switched B cells. 

### 4.2. Role of Mucosal Dendritic Cells and TLRs

Mucosal conditioning of DCs occurs via resident tissue-derived factors, such as thymic stromal lymphopoietin (TSLP), IFN-*β*, RA, and TGF-*β*, but also by ligation of toll-like receptor (TLR) ligands expressed by (commensal) bacteria [82–84] (Figure 2). Epithelial cells release these DC conditioning factors in addition to other IgA stimulatory factors in response to TLR ligands [85]. In the gut lamina propria, several specialized DC subsets are described with an enhanced intrinsic capacity to drive IgA CSR. For example, Tip DCs express inducible nitric oxide synthase (iNOS) in response to TLR signaling and initiate TI IgA production by releasing BAFF and APRIL [86]. CD11c^hi^CD11b^hi^ DCs induce TI IgA production upon sensing bacteria through TLR5, a process that elicits release of RA and IL-6 [87]. CD103^+^ DCs are known for driving the differentiation of FoxP3^+^ Treg cells. As the main RA producing subset, they are also responsible for imprinting gut-homing molecules on B cells and support IgA synthesis [74]. How these DCs acquire their tolerogenic properties is not yet fully understood, but a role for microbial activation was suggested [88, 89]. Recently, it was published that *in vitro* mouse CD11b^hi^ lung DCs induce IgA more efficiently than CD103^+^ lung DCs [90]. The use of CD11b as a marker for lung DCs is however confusing, as CD11b is not only found on a subset of conventional (c)DCs, but also on the population of monocyte-derived DCs (moDCs) that are recruited to the lungs at times of inflammation [91]. 

In addition to the conventional mouse DC subsets which drive IgA synthesis and are portrayed in the previous paragraph, also a plasmacytoid (p)DC subset has been described [92]. These pDCs differ from cDCs in expressing lower amounts of CD11c, yet they produce very high amounts of IFN*α*. Although both mouse gut lymph node-derived pDCs and cDCs were able to support B-cell IgA production, pDCs were more superior *in vitro* due to type 1 IFN dependent enhanced APRIL and BAFF expression [93]. Also in humans, IFN*α* producing pDCs seem to be more advanced in supporting B-cell proliferation and differentiation into antibody producing cells, including IgA, compared to myeloid DCs (mDC, grossly equivalent to mouse cDC subset) [94, 95]. 

Altogether DCs form a crucial cell type in the differentiation of IgA responses. Although by different mechanisms, both cDCs and pDCs can promote Ig responses, and their IgA inducing capacity can be enhanced by local factors produced by mucosal tissues as well as by (local) microbial products such as TLR ligands. 

### 4.3. Early Priming and the Microbiota

The establishment of commensal flora in the intestine, and most likely also the respiratory tract [96], starts at birth and is considered to be crucial for stimulating and directing the development of the host immune system. Animals raised under germ-free conditions have an undeveloped immune system with fewer germinal centers and decreased number of IgA-producing plasma cells [97]. Interestingly, gut microbiota is necessary for a protective immune system, including mucosal IgA responses, in the airways. In response to OVA, germ-free (GF) mice developed more severe features of airway inflammation compared to control specific pathogen free (SPF) mice, which could be reversed by recolonization of GF mice with complex commensal flora. Furthermore, the absence of commensal bacteria was associated with less pDCs and attenuated production of IgA in the airways [98]. Human studies have also suggested the link between commensals and allergy. Indeed, children who developed allergy had significantly less diverse gut microbiota and lower levels of salivary SIgA [99], while, intestinal colonization by *Staphylococcus aureus* was associated with high circulating IgA levels and with a lower frequency of eczema [100]. The exact number and diversity of an individual's community of commensals seem to be determined by factors occurring in early childhood [101]. Several mouse and human studies have shown that early life (prenatal, preconception) exposure to environments characterized by a diverse and concentrated microbial milieu such as traditional farming sites may protect against the development of allergic diseases [102–105]. Breast milk contains many Igs and may have a collective tolerogenic effect acting via sIgA, cytokines, and/or immune complexes [106]. 

A rich microbial environment contributes to mucosal tolerance and protective IgA responses, which are associated with protection against allergic asthma. The ideal candidate for an adjuvant stimulating protective IgA responses and thereby preventing development of allergic asthma could therefore be a microbial-derived molecule.

## 5. Strategy for Allergy Intervention via Induction of IgA 

### 5.1. Cholera Toxin B, an IgA Inducing Adjuvant

**Figure 3:**
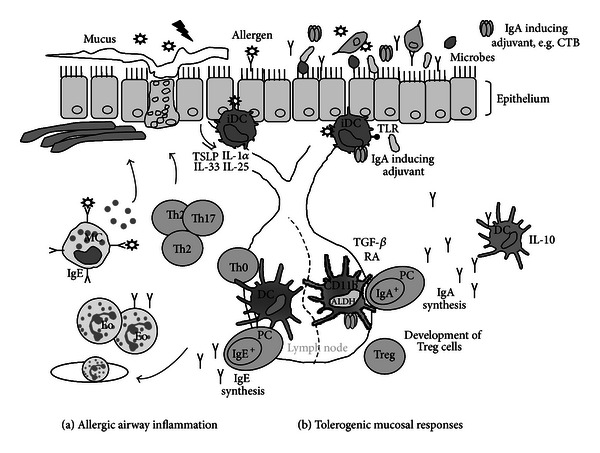
Crosstalk between epithelial cells and dendritic cells (DCs) in the lungs determines the balance between immunity and tolerance. (a) Allergen exposure in susceptible individuals may result in epithelial damage, initiating a cascade of immunological events leading to inflammation and allergic symptoms. (b) In contrast, IgA-inducing agents, such as CTB, alone or in combination with microbial exposure, prevent the development of allergic inflammation, by protecting the epithelial barrier and by effecting the function of various immune cells via DC. Immature DC (iDC), T helper 2 cell (Th2), T helper 17 cell (Th17), eosinophil (Eo), masT-Cell (MC), and plasma cell (PC).

Cholera toxin is the most widely experimentally used mucosal adjuvant, potentiating serum and local immune responses to coadministered antigens [107]. The enterotoxin Cholera Toxin is produced by the bacterium *Vibrio cholerae* and consists of an A and B subunit, each with distinct effects on cells of the immune system. The A subunit is known for its toxic (side) effects: after entering the cell cytosol, the A subunit triggers electrolyte efflux via activation of adenylate cyclase and increased cyclic AMP (cAMP) production, resulting in severe watery diarrhea. The B subunit of CT (CTB) is more considered as a nontoxic subunit, as it is not linked to the activation of cAMP and its adjuvant activity seems to be mainly associated with immunoregulatory events [107, 108]. For example, feeding of CTB conjugated to myelin basic protein before or after disease induction protected rats from experimental autoimmune encephalomyelitis [109], and nasal administration of CTB insulin significantly delayed incidence of spontaneous diabetes in NOD mice [110]. In these models, protection against autoimmunity by CTB/Ag conjugates was associated with the formation of Treg cells expressing IL-10 and/or TGF-*β* [109]. The tolerizing effect of CTB has also been shown to extend to other immune- mediated diseases. In a delayed type hypersensitivity model, (prolonged) oral treatment with low doses of OVA conjugated to CTB prevented sensitization and suppressed IgE antibody responses in sensitized mice [111]. Furthermore, intranasal pretreatment of CTB linked to the BetV1, a major allergen of birch pollen, prevented sensitization to the antigen by shifting the Th2 response towards Th1 and the induction of allergen-specific IgA responses [112]. Likewise, we found that CTB administration in the lungs stimulates local secretory IgA responses which protected against the development of allergic airway inflammation (AAI), while mice deficient for polymeric Ig receptor (pIgR) and lacking SIgA were not [113]. Interestingly, the primary action of CTB as an adjuvant may be primarily mediated through a direct effect on APCs such as DCs. Upon *in vivo* administration, CTB mainly effected cDCs and not so much pDCs, while adoptive transfer of *in vitro* generated CTB treated DCs was sufficient to enhance IgA responses in mouse lungs [113]. Furthermore, *in vitro* cocultures of CTB exposed bone marrow-derived DCs and B cells also resulted in the induction of IgA production. These *in vitro* experiments suggest that asynergism between CTB and MyD88-dependent TLR signals selectively imprints an IgA inducing phenotype in DCs, characterized by RALDH1 and TGF-*β* expression [114]. Upon exposure to CTB *in vivo*, ALDH activity was mainly enhanced in the CD11b^+^ DCs, which may include mo-DCs (Figure 3(b)). Also the experiments with the bone marrow-derived DCs differentiated in GM-CSF, which are a good model for these inflammatory DCs, may suggest that (CD11b^+^) moDCs could be responsible for CTB induced IgA responses in the mouse airways. This is certainly a possibility as CTB seems to work best at inducing IgA responses when accompanied by some degree of LPS. LPS is a known trigger of moDC recruitment [115]. Until we have more specific depleting antibodies or transgenic mouse strains to selectively deplete moDCs, we can however only speculate at this stage whether this is true.

If we are to exploit the full potential of IgA as an immunomodulatory immunoglobulin in allergic asthma and other immune mediated diseases, the role of different DC subsets in the regulation of humoral IgA responses and modulation by adjuvants should be studied in more detail.

### 5.2. Allergen-Specific Immunotherapy

Allergen-specific immunotherapy (SIT) represents the only curative treatment of allergic diseases currently available and involves the incremental delivery of the allergen to which the individual is sensitive [116]. Successful IT components of the regulatory network such as Treg cells and the cytokine IL-10 are elevated, while allergen-specific IgE levels are reduced. It is hypothesized that the enhanced immunoregulatory network is instrumental in suppressing allergen-specific effector T cells which are responsible for many of the characteristics of allergic diseases. IL-10 does not only contribute to T-Cell tolerance but also potently suppresses total and allergen-specific IgE, and it simultaneously increases IgG4 and IgA production in cultures [117, 118]. Interestingly, successful immunotherapy is also associated with increases in IgA responses *in vivo*. In a 2-year double blind trial, grass-pollen immunotherapy induced a shift in allergen-specific antibody response towards IgA2, which correlated with increased local TGF-*β* expression and induced monocyte IL-10 expression [119]. Another study using sublingual grass-pollen immunotherapy (SLIT) reported increases in allergen-specific IgG4 and IgA [120]. 

In its current form, SIT has major drawbacks and cannot compete with treatment on the basis of symptom relief (antihistamines and corticosteroids) for many asthma patients. High concentrations of allergen extract need to be administered on a long term (~5 years) and regular basis. This introduces a risk of potentially life-threatening allergic reactions [121]. Therefore, it might be interesting to apply the use of tolerogenic adjuvants, specifically inducing Treg cells and/or secretory IgA to improve efficacy and safety of SIT. For example, when the allergen is coupled to the adjuvant CTB, it will be efficiently targeted to the DC [113], allowing the use of lower allergen doses and decreasing the risk of anaphylactic shocks. Future experiments in mouse models for true allergens, like birch pollen or house-dust mite, experimental SIT models, and (cells from) allergic patients will need to point out the usefulness of application of the class of “mucosal” adjuvants in current SIT protocols. 

### 5.3. Boosting IgA as a Preventive Strategy

The establishment of commensal flora in the intestine and respiratory tract starts at birth and is considered to be crucial for stimulating and directing the development of the host immune system, including the mucosal IgA response [97, 122, 123]. In our *in vitro* coculture system, we confirmed the role for microbial-derived TLR ligands in the conditioning of DCs for stimulating IgA responses. Interestingly, the mucosal adjuvant CTB does not only enhance IgA induction by TLR-ligand primed DCs, but also initiates IgA production in the case of low dose exposure to MyD88-activating signals which are insufficient to induce IgA on their own [114]. This is interesting considering the hypothesis that decreased or altered microbial exposure associated with an affluent life style is contributing to the increase in asthma prevalence during the last decades. Only recently we have started to appreciate the importance of the microbiota on human health, and restoring or manipulating disrupted host-microbiota relationship has become a potent strategy for treating inflammatory diseases, including asthma [124]. CTB could contribute to broad antibody repertoire and sufficient mucosal IgA levels in people with impaired or delayed IgA synthesis, by reducing the threshold for microbial signals or providing the necessary cosignals, to maintain mucosal immunity and local homeostasis (Figure 3). 

It was shown that children who developed allergy had less diverse (gut and airway) microbiota [99] and decreased serum or mucosal IgA responses [45, 46, 100] compared to healthy controls. Studies that measured IgA levels at different time points showed an increase over time which may be due to microbial exposure and microbiota development [45, 100]. Especially during the first months and year, events such as mode of birth delivery, type of “first” milk (breast versus formula milk), and microbial exposure will determine the composition of microbiota [122]. This suggests that particularly during this early period in life impaired IgA responses may allow for sensitization and/or development of allergic symptoms. By the time that allergic (asthma) patients have reached adulthood, impaired IgA responses may be restored to normal levels, but the “damage” has already occurred and allergen-specific inflammatory responses have developed. Indeed, in a cohort of adult allergic asthmatic patients, we did not find reduced (secretory) IgA levels in nasal washes compared to nonallergic controls (Gloudemans et al., unpublished observations). Alternatively, in a fraction of the allergic infants with a slowly developing mucosal IgA repertoire, allergy symptoms may relieve together with the establishment of a fully developed IgA response. Thus, one should keep in mind that the association between IgA and asthma may be misinterpreted using an adult cohort. 

## 6. Words of Caution 

The effector function of IgA is very much depending on local (inflammatory) factors present and needs to be carefully examined before applying IgA enhancing therapy. For example, IgA immune complexes bind with stronger affinity, subsequently resulting in cell activation and elimination of the pathogen [52, 53]. In certain cases, IgA complexes can even cause severe inflammation and pathology, like in immune complex glomerulonephritis [68, 125]. Importantly, eosinophils and neutrophils express receptors for IgA that can activate the cells upon binding of IgA immune complexes [51, 126], resulting in activation and/or degranulation of the cell [50, 127]. Therefore, also in severe asthma, where in addition to eosinophils also neutrophils are important mediators, IgA may aggravate the inflammation instead of promoting tolerance. Interestingly, both in patients with IgA nephropathy and in patients with asthma, abnormal glycosylation of the IgA antibody or the Fc*α*RI receptor was found, which may allow exacerbated immune responses and disease development instead [128, 129]. 

In addition to the isoform of IgA and local tissue factors, reactivity or specificity of the antibody will determine the receptor binding affinity and thus the immunological effect of Ig-receptor ligation. Primitive or polyreactive (natural) IgA antibodies are sufficient to protect the host from excess mucosal immune stimulation by harmless commensal bacteria and may protect against some noninvasive parasites [130, 131]. However, affinity maturation of IgA is necessary to provide protection from more invasive commensal bacteria and from true pathogens. Thus, there seems to be a correlation between the “sophistication” of the IgA response and the aggressiveness of the subsequent immune response, at least in the gut [132]. However, it still remains unclear how this applies for environmental particles in the lung, such as inhaled allergens. Therefore, to evaluate the efficacy of IgA-based treatment against allergic diseases, not only the level of mucosal IgA responses need to be carefully studied in health and disease, but also aspects such as the affinity and reactivity of the antibodies should be taken into account. 

Although local IgA induction during specific immunotherapy may have potential to improve the treatment of allergic airway inflammation, based on the dynamics of the development of IgA responses in life and the functional duality of the IgA-receptor interaction, it seems essential to stimulate IgA responses under noninflammatory conditions. Therefore, we hypothesize that the development of protective mucosal IgA responses will occur best in the context of a homeostatic environment, through the activation of dedicated mDCs and/or mo-DCs, aiming at the induction of a fully developed mucosal IgA repertoire in time and preventing the development of inflammatory responses to allergens (Figure 3).
